# Supplementary material for: Can women’s 3E index impede short birth interval? evidence from Bangladesh Demographic and Health Survey, 2017–18
Source: PLoS One. 2022 Jan 26;17(1):e0263003. doi: 10.1371/journal.pone.0263003 (PMC8791508; doi:10.1371/journal.pone.0263003)
Supplement: Attachment — Submitted filename: PONE-D-21-15599_reviewer final.docx [file pone.0263003.s002.docx]

PLOS ONE

**Can Women’s 3E Index Impede Short Birth Interval? Evidence from Bangladesh Demographic and Health Survey, 2017-18**

--Manuscript Draft--

| **Manuscript Number:** | PONE-D-21-15599 |
| --- | --- |
| **Article Type:** | Research Article |
| **Full Title:** | Can Women’s 3E Index Impede Short Birth Interval? Evidence from Bangladesh Demographic and Health Survey, 2017-18 |
| **Short Title:** | Role of Women’s 3E on Short Birth Interval in Bangladesh |
| **Corresponding Author:** | Fatima Tuz Zahura, MS  University of Dhaka: Dhaka University Dhaka, BANGLADESH |
| **Keywords:** | Keywords: Short Birth Interval, Education, Economic Status, Empowerment, Women's 3E Score |
| **Abstract:** | Background  Women’s empowerment, education and economic status are jointly introduced as women’s 3E. A number of studies found the significant association of women’s 3E in maternal health outcomes, but no studies, to the best of knowledge, have been found to justify the joint influence of women’s 3E on the birth interval. As several studies have revealed that the short birth interval increases the risk of adverse maternal, perinatal and infant outcomes and it is also responsible for increasing the country’s population size, more research is needed on the birth interval. Therefore, the present study aimed to investigate the influence of women’s 3E on the short birth interval after controlling the other selected covariates.  Methods  Data from the Bangladesh Demographic and Health Survey (BDHS), 2017-18 have been used to serve the purpose of the study. To measure the birth interval, at least two live births for non-pregnant mothers and at least one live birth for currently pregnant mothers born in the 5 years before the survey were included in the study. The Chi- Square test was applied to know the unadjusted association of the selected covariates including women’s 3E with the short birth interval. In order to find out the adjusted association of women’s 3E with the short birth interval, sequential binary logistic regression models have been used.  Results  The study found that about 23% of births in Bangladesh were born in a short birth interval. The likelihood of subsequent births of women decreases with an increase in the score of women’s 3E before or after controlling the characteristics of women, child and households. The results of the final model show that mothers with the coverage of 50% - 75%, 75% - 100% and full coverage (100%) in 3E have a 23%, 41% and 42% lower odds of having short birth interval compared to mothers with coverage of below 50% in 3E, respectively.  Conclusion  Bangladesh still lags behind in meeting the minimum requirements for inter-birth intervals set by the World Health Organization. The study has shown that the 3E in women can contribute in prolonging the duration of subsequent births in Bangladesh. Policy-making interventions are needed to raise awareness among uneducated, under- empowered and economically poor reproductive women through family planning and fertility control programs so that the country can achieve the desired fertility rate. |
| **Order of Authors:** | Fatima Tuz Zahura, M.S. |
|  | Kanchan Kumar Sen |
|  | Shahnaz Nilima |
|  | Wasimul Bari |
| **Additional Information:** |  |

| **Question** | **Response** |
| --- | --- |
| **Financial Disclosure**  Enter a financial disclosure statement that describes the sources of funding for the work included in this submission. Review the [submission guidelines](http://journals.plos.org/plosone/s/submission-guidelines#loc-financial-disclosure-statement) for detailed requirements. View published research articles from [*PLOS ONE*](http://journals.plos.org/plosone/) for specific examples.  This statement is required for submission and **will appear in the published article** if the submission is accepted. Please make sure it is accurate. | The author(s) received no specific funding for this work. |
| **Unfunded studies**  Enter: *The author(s) received no specific funding for this work.*  **Funded studies**  Enter a statement with the following details:   - Initials of the authors who received each award - Grant numbers awarded to each author - The full name of each funder - URL of each funder website - Did the sponsors or funders play any role in the study design, data collection and analysis, decision to publish, or preparation of the manuscript? - **NO** - Include this sentence at the end of your statement: *The funders had no role in study design, data collection and analysis, decision to publish, or preparation of the manuscript.* - **YES** - Specify the role(s) played. |  |
| * typeset |  |
| **Competing Interests**  Use the instructions below to enter a competing interest statement for this submission. On behalf of all authors, disclose any [competing interests](http://journals.plos.org/plosone/s/competing-interests) that could be perceived to bias this work—acknowledging all financial support and any other relevant financial or non- financial competing interests. | The authors have declared that no competing interests exist. |

| This statement is required for submission and **will appear in the published article** if the submission is accepted. Please make sure it is accurate and that any funding sources listed in your Funding Information later in the submission form are also declared in your Financial Disclosure statement.  View published research articles from  [*PLOS ONE*](http://journals.plos.org/plosone/) for specific examples. |  |
| --- | --- |
| **NO authors have competing interests**  Enter: *The authors have declared that no competing interests exist.*  **Authors with competing interests**  Enter competing interest details beginning with this statement:  *I have read the journal's policy and the authors of this manuscript have the following competing interests: [insert competing interests here]* |  |
| * typeset |  |
| **Ethics Statement**  Enter an ethics statement for this submission. This statement is required if the study involved:   - Human participants - Human specimens or tissue - Vertebrate animals or cephalopods - Vertebrate embryos or tissues - Field research   Write "N/A" if the submission does not require an ethics statement.  General guidance is provided below. Consult the [submission guidelines](http://journals.plos.org/plosone/s/submission-guidelines#loc-guidelines-for-specific-study-types) for detailed instructions. **Make sure that all information entered here is included in the Methods section of the manuscript.** | N/A |

| **Format for specific study types**  **Human Subject Research (involving human participants and/or tissue)**   - Give the name of the institutional review board or ethics committee that approved the study - Include the approval number and/or a statement indicating approval of this research - Indicate the form of consent obtained (written/oral) or the reason that consent was not obtained (e.g. the data were analyzed anonymously)   **Animal Research (involving vertebrate animals, embryos or tissues)**   - Provide the name of the Institutional Animal Care and Use Committee (IACUC) or other relevant ethics board that reviewed the study protocol, and indicate whether they approved this research or granted a formal waiver of ethical approval - Include an approval number if one was obtained - If the study involved *non-human primates*, add *additional details* about animal welfare and steps taken to ameliorate suffering - If anesthesia, euthanasia, or any kind of animal sacrifice is part of the study, include briefly which substances and/or methods were applied   **Field Research**  Include the following details if this study involves the collection of plant, animal, or other materials from a natural setting:   - Field permit number - Name of the institution or relevant body that granted permission |  |
| --- | --- |
|  |  |
| **Data Availability**  Authors are required to make all data underlying the findings described fully available, without restriction, and from the time of publication. PLOS allows rare exceptions to address legal and ethical concerns. See the [PLOS Data Policy](http://journals.plos.org/plosone/s/data-availability) and [FAQ](http://journals.plos.org/plosone/s/data-availability#loc-faqs-for-data-policy) for detailed information. | Yes - all data are fully available without restriction |

| A Data Availability Statement describing where the data can be found is required at submission. Your answers to this question constitute the Data Availability Statement and **will be published in the article**, if accepted. |  |
| --- | --- |
| **Important:** Stating ‘data available on request from the author’ is not sufficient. If your data are only available upon request, select ‘No’ for the first question and explain your exceptional situation in the text box. |  |
| Do the authors confirm that all data underlying the findings described in their manuscript are fully available without restriction? |  |
| **Describe where the data may be found in full sentences. If you are copying our sample text, replace any instances of XXX with the appropriate details.**   - If the data are **held or will be held in a public repository**, include URLs, accession numbers or DOIs. If this information will only be available after acceptance, indicate this by ticking the box below. For example: *All XXX files are available from the XXX database (accession number(s) XXX, XXX.*). - If the data are all contained **within the manuscript and/or Supporting Information files**, enter the following: *All relevant data are within the manuscript and its Supporting Information files.* - If neither of these applies but you are able to provide **details of access elsewhere**, with or without limitations, please do so. For example:   *Data cannot be shared publicly because of [XXX]. Data are available from the XXX Institutional Data Access / Ethics Committee (contact via XXX) for researchers who meet the criteria for access to confidential data.*  *The data underlying the results presented in the study are available* | Data underlying this study can be found at https://dhsprogram.com after completing a registration process and sending a request in the website. |

| *from (include the name of the third party and contact information or URL).*   - This text is appropriate if the data are owned by a third party and authors do not have permission to share the data.   * typeset |  |
| --- | --- |
| Additional data availability information: |  |

Manuscript	[Click here to access/download;Manuscript;Manuscript.short birth interval.docx](https://www.editorialmanager.com/pone/download.aspx?id=28988626&guid=465a756a-6857-4db3-8d6c-c796a95eb85a&scheme=1)

# Title

Can Women’s 3E Index Impede Short Birth Interval? Evidence from Bangladesh Demographic and Health Survey, 2017-18

# Short title

Role of Women’s 3E on Short Birth Interval in Bangladesh

# Authors

1. Fatima-Tuz-Zahura (Corresponding author)

Email: [zahura.fatima@](mailto:zahura.fatima@yahoo.com)du.ac.bd

2. Kanchan Kumar Sen Email: [kksen.sta@du.ac.bd](mailto:kksen.sta@du.ac.bd)
3. Shahnaz Nilima Email: [s.nilima@du.ac.bd](mailto:s.nilima@du.ac.bd)
4. Wasimul Bari Email: [w_bari@du.ac.bd](mailto:w_bari@du.ac.bd)

Address^1-4^: Department of Statistics, University of Dhaka, Dhaka-1000, Bangladesh

# 1 Abstract

2 **Background**
3 Women’s empowerment, education and economic status are jointly introduced as women’s 3E. A
4 number of studies found the significant association of women’s 3E in maternal health outcomes,
5 but no studies, to the best of knowledge, have been found to justify the joint influence of women’s
6 3E on the birth interval. As several studies have revealed that the short birth interval increases the
7 risk of adverse maternal, perinatal and infant outcomes and it is also responsible for increasing the
8 country’s population size, more research is needed on the birth interval. Therefore, the present
9 study aimed to investigate the influence of women’s 3E on the short birth interval after controlling
10 the other selected covariates.

# 11 Methods

12 Data from the Bangladesh Demographic and Health Survey (BDHS), 2017-18 have been used to
13 serve the purpose of the study. To measure the birth interval, at least two live births for non-
14 pregnant mothers and at least one live birth for currently pregnant mothers born in the 5 years
15 before the survey were included in the study. The Chi-Square test was applied to know the
16 unadjusted association of the selected covariates including women’s 3E with the short birth
17 interval. In order to find out the adjusted association of women’s 3E with the short birth interval,
18 sequential binary logistic regression models have been used.

19

# 20 Results

21 The study found that about 23% of births in Bangladesh were born in a short birth interval. The
22 likelihood of subsequent births of women decreases with an increase in the score of women’s 3E
23 before or after controlling the characteristics of women, child and households. The results of the
24 final model show that mothers with the coverage of 50% - 75%, 75% - 100% and full coverage
25 (100%) in 3E have a 23%, 41% and 42% lower odds of having short birth interval compared to
26 mothers with coverage of below 50% in 3E, respectively.

# 27 Conclusion

28 Bangladesh still lags behind in meeting the minimum requirements for inter-birth intervals set by
29 the World Health Organization. The study has shown that the 3E in women can contribute in
30 prolonging the duration of subsequent births in Bangladesh. Policy-making interventions are
31 needed to raise awareness among uneducated, under-empowered and economically poor
32 reproductive women through family planning and fertility control programs so that the country can
33 achieve the desired fertility rate.

# 34 Keywords: Short Birth Interval, Education, Economic Status, Empowerment, Women’s 3E

35 **Score**

36

37

38

39

# 40 Introduction

41 Birth interval, defined as the length of two consecutive live births, plays an important role in
42 changing the structure of the population of a country. Moreover, health promotion policies for
43 women and children are mainly governed by the birth spacing. The World Health Organization
44 (WHO) technical consultation on birth spacing has recommended a minimum of 33 months
45 between two consecutive live births or 24 months for birth to conception of a pregnancy to reduce
46 the risk of adverse maternal, perinatal and infant health outcomes [1-4]. A short birth interval,
47 defined as the interval below the WHO recommendation, is responsible for an increased risk of
48 anemia, malnutrition, toxemia, uterine rupture and self-induced abortion, gestational diabetes,
49 placental bleeding, and even maternal mortality [2, 5-13]. It has an adverse impact on neonatal and
50 child health such as preterm births, low birth weights, infant mortality, and it also affects their
51 standard physical and intellectual growth [8, 9, 14-23]. Several studies have shown that babies
52 born at least two years apart are more likely to survive and have a lower risk of premature birth,
53 low birth weight and malnutrition [21, 24]. In addition, a study conducted for developing countries
54 revealed that babies born at an optimum birth interval of three to five years after their previous
55 siblings are about 2.5 times likely to survive compared to babies born at below two-year interval
56 [3]. Studies found that an estimated 2.6 million stillbirths occur each year, and most stillbirths
57 during pregnancy can be prevented by optimizing the birth interval [25-27]. Again, an optimum
58 interval can also create positive health outcomes for mothers, where mothers can get recovery
59 support from macro and micronutrient depletion that occurs during pregnancy and lactation [8, 24,
60 28]. Therefore, an optimal birth interval is required for the protection of both mother and babies

61	[29-31].

62 Birth interval is a major indicator of fertility and it may have an influence in reducing the
63 size of the country's population through fertility control [2, 32-34]. As fertility rate of a country is
64 inextricably related to the role of women, the status of women consisting of several indicators
65 undoubtedly influences the population growth of a country. Several studies have shown that
66 maternal education has a positive relationship with a country's population size [35-37]. Moreover,
67 the economic status of women is also related to fertility; the higher the economic status of women,
68 the lower the fertility rate [38]. However, women empowerment could be another pathway to
69 assess fertility preferences because empowered women can enhance their ability by expanding
70 resources that can have control over achieving their ideal number of children [39, 40]. Women’s
71 education, empowerment, and economic status jointly introduced as the women’s 3E had already
72 been proven to influence maternal health care utilization in developing countries [41, 42] and to
73 be one of the major determinants to boost up vaccination uptakes in Pakistan [43]. A study
74 conducted in Bangladesh found a significant association between number of living children and
75 status of women namely level of education, occupation, and having discussion with their partners
76 regarding family planning issues [44]. In this study, women’s 3E have been explored under the
77 area of fertility to investigate the direction and magnitude of their association with birth interval.
78 A study using pooled data obtained from three Bangladesh Demographic and Health
79 Surveys (BDHS) of the years 2004, 2007, and 2011 data, revealed that parity, survival status of
80 index child, father’s education, wealth quintile, place of residence, division, and year of the survey
81 had significant effects on birth interval [45]. Research work conducted in the country analyzed
82 determinants of birth interval using BDHS, 2007 data and showed that previous birth interval,
83 mother’s education, working status, and mass media exposure had a significant association with
84 birth intervals [46]. Another study conducted by Mahmood and Zainab, utilizing the BDHS 2004
85 data, found that mother’s education and place of residence had a great influence on variation in
86 birth intervals in Bangladesh [47]. However, a study in the Philippines revealed a strong positive
87 association between longer birth interval and women’s decision-making autonomy [48].
88 Addressing a number of social needs, the Sustainable Development Goals (SDGs) aim to
89 ensure a peaceful, sustainable, prosperous and equitable life for all in the world by 2030. Women’s
90 3E is directly linked to SDG1 for poverty alleviation, SDG4 for ensuring equitable quality
91 education, and SDG5 for women’s empowerment and gender equality [49]. Again, women’s
92 empowerment, education, and economic status can make a significant contribution to the
93 achievement of many other SDGs, and therefore it is time to address these factors for maternal and
94 child health outcomes in achieving the SDGs. According to the report of the World Bank, it was
95 demonstrated that global development would be attained through gender equality [50]. Therefore,
96 it is very crucial to study the importance of women’s 3E in different aspects that can help
97 policymakers to build up new strategies to ensure economic growth by promoting gender equity.
98 Although the total fertility rate (TFR) in Bangladesh has been 2.3 births per woman since
99 2011, the median birth interval has increased from 47.4 to 55.7 months over the last seven years
100 [51, 52]. Despite this increase, TFR is still far from the goal of the health sector program to reach
101 the target of 2 births per woman by 2022 [51]. Again, approximately 32%, 29%, and 25% of births
102 were born at below three years of birth spacing in 2011, 2014, and 2017-18, respectively, where
103 the results indicate a slow decline in the overall prevalence of short birth intervals [51-53].
104 Therefore, Bangladesh is still facing the problem of birth spacing to achieve the WHO
105 recommendation that every mother should give subsequent birth at or after 33 months. Hence, it
106 is necessary to accelerate the duration of birth interval in Bangladesh through necessary policy-
107 making measures. Since birth interval can affect fertility, maternal and child health, it is a matter
108 of great concern for researchers and policy makers to study the factors that affect birth interval in
109 overpopulated countries like Bangladesh. The current study hypothesizes that 3E of women may
110 be a necessary target for policy-making interventions to reduce subsequent births at short interval
111 so that the country can achieve the desired fertility rate.
112 In this study, an attempt has been made to investigate the association of women’s 3E with
113 short birth interval in Bangladesh controlling other important variables related to women, child,
114 and household characteristics. To the researchers’ knowledge, no study has been conducted in
115 Bangladesh on this topic so far. For the purpose of analysis, secondary data extracted from BDHS,
116 2017-18 has been analyzed using the sequential approach of the logistic regression model.

# 118 Materials and Methods

120 *Study Design*
121 The study utilized the birth interval data extracted from the nationally representative Bangladesh
122 Demographic and Health Survey (BDHS), 2017-18 data. The survey had been conducted
123 following a two-stage stratified sample of households. In the first stage, the survey selected 675
124 enumeration areas (EAs), 227 EAs in urban areas, and 448 in rural areas, with probability
125 proportional to EA size and with an independent selection within each stratum. Afterward, 30
126 households from each of the selected EAs have been chosen in the second stage resulting in a total
127 of 20,250 residential households, but a total of 20,160 households were selected for the survey in
128 672 clusters after elimination of three clusters that were completely eroded by floodwater. Finally,
129 BDHS successfully interviewed 20,127 ever-married women aged 15-49 years on a complete
130 history of their live births along with some important socio-economic and demographic
131 characteristics.
133 *Participants*
134 To get information for the current study, at least one birth for current pregnant mothers and at least
135 two births for non-pregnant mothers born in the 5 years before the survey have been considered
136 which results in a total of 5,441 births. The selected births have come from a total of 4,866
137 reproductive women in the study. The details of sample selection are given in Figure 1. It is noted
138 that the current pregnancies were treated as births and duration between last birth and initiation of
139 pregnancy is considered as birth interval.

# 141 [Figure 1 here]

143 *Outcome measure*
144 The current study aimed to measure the association of women’s 3E with short birth interval in
145 Bangladesh. Therefore, the short birth interval was considered as outcome variable in the study,
146 where it was defined as the interval of below 33 months between two consecutive live births or
147 fewer than 24 months for last birth to conception of a pregnancy following WHO guidelines on
148 birth spacing [1].
149 To compute the birth intervals from the data extracted from BDHS, 2017-18, following
150 two steps have been considered:
151 (i)	Currently non-pregnant mothers having at least two live births born in the preceding five
152 years of survey were selected. Then birth intervals were measured taking the duration
153 (in months) between two consecutive live births.
154 (ii)	Currently pregnant mothers having at least one live birth born in preceding five years of
155 survey were selected. To include the birth interval for last birth to current pregnancy, the
156 intervals were computed taking the difference between the current age of last child (in
157 months) and the duration of current pregnancies (in months) and for all other births birth
158 intervals are computed by taking duration (in months) between two successive births.
159 *Main explanatory variable: Women’s 3E Index*
160 This study emphasized understanding the short birth interval with respect to women’s coverage in
161 3E. Women’s empowerment, education and economic status were jointly treated as women’s 3E.
162 Women’s empowerment cannot be measured directly due to its latent phenomena. As a result,
163 different sets of indicators have been used in different studies to define women’s empowerment.
164 Among those different indicators, women’s participation in household decision making is
165 estimated to be the most common that has been used in 37 studies during the year 1990 to 2012
166 [54]. In this study, a women’s empowerment has been assessed using the variables regarding their
167 participation in household decision making, available in the DHS survey. These variables include
168 respondent’s involvement in decisions on (i) spending her own earning, (ii) spending her partner’s
169 earning, (iii) her own health care, (iv) large household purchases, and (v) visits to family relatives.
170 Finally, these five variables are converted into dichotomous variables, where a value of 1 is given
171 among those women who decided alone or in partnership with their husbands/partners, and a value
172 of 0 for otherwise. Again, the principal component analysis (PCA) has been applied to create the
173 wealth score index using the available household assets set by the survey, and then the score is
174 divided into two equal parts based on median to define economic status as poor and rich. Therefore,
175 the economic status is an indicator variable giving a value of 1 for rich and 0 for poor women. The
176 women’s education is recategorized into two categories to define indicator variable: uneducated
177 (no and primary) coded as 0 and educated (secondary and higher) coded as 1. To obtain the joint
178 influence of empowerment, education and economic status, women’s 3E index has been measured
179 by computing the mean score of the indicator variables following WHO approach [55]. The
180 approach follows a rule to give equal weights in each domain (empowerment, education and
181 economic status), and also to give equal weights in each indicator variables of the weighted
182 domains to calculate the total mean score. For clear understanding, mathematically it can be written
183 as

𝑘

1

𝑍_𝑖_ = ( ∑

ℎ𝑗

1

∑ 𝑥𝑖𝑗𝑙

) × 100 ; 𝑖 = 1, 2, … , 𝑛; 𝑗 = 1, 2, … , 𝑘; 𝑙 = 1, 2, … , ℎ_𝑗_,

𝑘

𝑗=1

ℎ_𝑗_

𝑙=1

185 where 𝑍_𝑖_ is the total mean score of 𝑖^𝑡ℎ^case, 𝑘 is the number of domains, ℎ_𝑗_is the number of
186 indicators in 𝑗^𝑡ℎ^domain, 𝑥_𝑖𝑗𝑙_ is the value of 𝑙^𝑡ℎ^indicator in 𝑗^𝑡ℎ^ domain for 𝑖^𝑡ℎ^case, and 𝑛 is the total
187 number of cases [55, 56]. In the study, there are five indicators for empowerment and one for both
188 education and economic status to calculate the total mean, which is the score of women’s 3E for
189 each woman. In the study, the scale reliability coefficient (Cronbach’s 𝛼) has also been calculated,
190 and it was found to be of 0.73. Therefore, the result indicates that the selected seven indicators are
191 internally related to each other and hence there is no problem in creating a score index [57]. To
192 explore the effect of women’s 3E on birth spacing at different levels of the score, we classified the
193 score into four categories as below 50%, 50% - 75%, 75% - 100%, and full coverage in 3E (100%).
194 Under full coverage of women’s 3E, a woman is fully empowered, educated and economically
195 prosperous.
196 *Covariates*
197 Based on a number of literatures [2, 20, 45-48, 58-68], the control variables chosen in this study
198 are women related variables: age at marriage, access to media, employment status, husband’s
199 education and experience of terminated pregnancy; preceding child related variables: gender, birth
200 order and survival status; household related variables: migration status, region and place of
201 residence. The definition and measurements of the variables are given in Table 1.

# 202 Table 1. Definition and measurements of the variables used in the study based on the 2017-

203 **18 BDHS**

| **Characteristics** | **Variables** | **Measurement** |
| --- | --- | --- |
| Birth Spacing | Birth Interval | Duration of months between two successive live births or birth  to successive pregnancy |
|  | Short Birth Interval | Birth Interval of below 24 months for birth-to-pregnancy or  fewer than 33 months for birth-to-birth interval (Categories: Yes/No) |
| **Women’s 3E Index | Women’s  Empowerment | Women’s empowerment has been assessed using the variables  regarding their participation in household decisions. |
|  | Women’s Education | The survey created four categories of women’s education: no, primary, secondary, and higher. In the study, the education is converted into two categories: uneducated (no and primary) and  educated (secondary and higher) |
|  | Economic Status | The principal component analysis (PCA) has been applied to create the wealth score index using the available household assets set by the survey, and then the score is divided into two equal parts based on median to define economic status as poor  and rich. |
| Women | Husband’s Education | Following the conversion of women’s education, the current  study also created the husband’s education in two categories: uneducated and educated |
|  | Maternal Age at  Marriage | Age at marriage is categorized into three categories as below 15  years, 15-17, and 18 or 18+ years. |
|  | Pregnancy Termination | Pregnancy termination is defined as ending of a pregnancy which results in no live birth. The study used this variable as whether the women ever terminated a pregnancy or not before  the survey. |
|  | Employment  Status | Yes/No |
|  | Media Exposure | Media exposure is created based on variables of watching TV, listening radio, and reading newspapers/magazine, and a woman is exposed to media who has exposure to either of the  media sources (Categories: Yes/No) |
|  | Religion | The original dataset has four categories of religion: Islam,  Hinduism, Buddhism, and Christianity, but the religion has |

|  |  | been recategorized into two categories as Muslim (Islam) and  Non-Muslim (Hinduism, Buddhism, and Christianity) in the study. |
| --- | --- | --- |
|  | Intimate Partner Violence | Intimate partner violence has been measured using the five proxy variables of wife beating: (i) beating justified if wife goes out without telling husband (ii) beating justified if wife neglects the children (iii) beating justified if wife argues with husband  (iv) beating justified if wife refuses to have sex with husband and (v) beating justified if wife burns the food. A woman is treated as violated by her partner/husband if she faces in any of  the above beatings. Categories: Yes/NO |
| Child | Gender of  Preceding Child | Gender is considered for preceding Child in the study.  Categories: Male/Female |
|  | Birth Order of  Preceding Child | Categorized into three categories as first birth, second birth, and  third/higher birth |
|  | Survival of  Preceding Child | Yes/No |
| Household | Place of  Residence | Urban/rural |
|  | Region | The original dataset has eight divisions, but the study divided whole Bangladesh into three regions using divisions: Central (Barisal, Mymensingh and Dhaka divisions), Eastern (Chattogram and Sylhet divisions), and Western (Khulna,  Rajshahi and Rangpur divisions). |
|  | Migration Status | Yes/No |

*Note*. **Women’s 3E index has been measured using the variables of women’s empowerment, education and economic status, where the score of women’s 3E is computed following WHO approach [55]

205 *Statistical Analysis*
206 To get the univariate results for the selected variables, the frequency percentage has been used in
207 The study. In bivariate analysis, the chi-square test with cross-tabulation was used to evaluate the
208 Unadjusted association between the selected variables and the short birth interval. To find the
209 adjusted association of woman’s 3E with short birth interval, a sequential approach of logistic
210 regression model is used: (a) Model I: classified mean score of women’s 3E; (b) Model II: Model
211 I + women related variables; (c) Model III: Model II + preceding child related variables; and (d)
212 Model IV: Model III + household related variables. Covariates having p-value less than 0.2 in the
213 bivariate analysis were considered in the regression models.

# 215 Results

216 **Descriptive Statistics**
217 Table 2 illustrates the frequency percentage and mean score of the indicators of women’s 3E.
218 Among the indicators of women's empowerment, a high proportion in visiting family relatives,
219 taking their own health care, and a low proportion in spending their own earnings were found.
220 More than one-third (35.31%) of selected women can decide on their own earnings, and about
221 three-fourth can take the decisions on their own health care (75.83%) or family visits (74.31%)
222 alone or jointly with their husbands. The results imply that most Bangladeshi women are
223 empowered to make their own healthcare decisions, but women are less empowered to make
224 decision in spending their own earnings. Again, at present, more than 50 percent of Bangladeshi
225 women are educated and about 50% of women fail in the rich economic category. To compute
226 the score of women’s 3E, three domains are considered, which are women’s empowerment
227 consisting of five indicators, education and economic status. The mean score for these domains
228 were found to be 21.8, 18.8, and 16.7, respectively. Hence the total mean score of women’s 3E
229 were 57.3. It indicates that on the average, a woman still requires 42.7 points to reach the full
230 coverage of women’s 3E.

# 233 Table 2. Frequency distribution of several indicators of women’s 3E along with its mean score and 95% confidence intervals

**Indicators in computing women’s 3E**

***Women’s empowerment	Education Status**

**Economic Status**

**Mean score of women’s**

|  | *Decided on spending her own earnings* | *Decided on spending her partner’s*  *earnings* | *Decided on her own health care* | *Decided on large household purchases* | *Decided on visits to family relatives* | *Educated* | *Rich* | **3E** |
| --- | --- | --- | --- | --- | --- | --- | --- | --- |
| **Percentage** | 35.31 | 69.16 | 75.83 | 72.74 | 74.31 | 56.39 | 49.97 |  |
| **Mean score (in %)** | 2.4 | 4.6 | 5.1 | 4.8 | 5.0 | 18.8 | 16.7 |  |
|  | [2.3-2.4] | [4.5-4.7] | [5.0-5.1] | [4.8-4.9] | [4.9-5.0] | [18.4-19.2] | [16.2-17.1] |  |
| **Total** |  |  | **21.8** |  |  | **18.8** | **16.7** | **57.3** |
|  |  |  | **[21.5-22.1]** |  |  | **[18.4-19.2]** | **[16.2-17.1]** | **[56.5-58.0]** |

234 *Note*: ***** Women participated in household decisions alone or in partnership with their husbands/partners
237 Basic background characteristics of the respondents along with the percentage frequency
238 distributions have been presented in Table 3. Table 3 also displays the percentage of short birth
239 interval in each category of selected variables with 95% confidence interval as well as p-values
240 obtained from chi-square test which signifies the unadjusted association of the variables with
241 short birth interval.

# 242 Table 3. Frequency percentage, and short birth interval with 95% confidence intervals (CI)

243 **by the selected background characteristics**

| **Characteristics** | **Frequency** | **Short Birth Interval** | **p-value** |
| --- | --- | --- | --- |
|  | **Percentage** | **(95% CI)** |  |
| **Women’s 3E** |  |  |  |
| Coverage of < 50% | 39.1 | 27.1 [25.2-28.9] |  |
| 50% - 75% | 30.9 | 21.4 [19.5-23.4] | <0.001 |
| 75% - 100% | 23.9 | 18.3 [16.2-20.1] |  |
| Full Coverage (100%) | 6.0 | 14.9 [11.1-18.8] |  |
| **Husband’s Education** |  |  |  |
| Uneducated | 56.5 | 24.3 [22.7-25.8] | <0.001 |
| Educated | 43.5 | 20.2 [18.6-21.8] |  |
| **Maternal Age at Marriage** (in |  |  |  |
| years) |  |  |  |
| 10 - 15 | 28.0 | 19.1 [17.1-21.0] |  |
| 15 - 18 | 44.9 | 22.5 [20.8-24.1] | <0.001 |
| 18+ | 27.1 | 26.1 [23.9-28.4] |  |
| **Pregnancy Termination** |  |  |  |
| No | 79.2 | 23.1 [22.8-24.3] | 0.053 |
| Yes | 20.8 | 20.4 [18.0-22.7] |  |
| **Media Exposure** |  |  |  |
| No | 40.2 | 25.0 [23.2-26.8] | <0.001 |
| Yes | 59.8 | 20.8 [19.4-22.2] |  |
| **Religion** |  |  |  |
| Muslim | 92.6 | 23.8 [21.6-23.9] | 0.111 |
| Non-Muslim | 7.4 | 19.3 [15.4-23.2] |  |
| **Intimate Partner Violence** |  |  |  |
| No | 80.8 | 22.4 [21.2-23.6] | 0.761 |
| Yes | 19.2 | 22.8 [20.3-25.4] |  |
| **Employment Status** |  |  |  |
| No | 54.8 | 24.8 [23.3-26.4] | <0.001 |

|  | Yes  **Gender of Preceding Child** | 45.2 | 19.7 [18.1-21.2] |  |
| --- | --- | --- | --- | --- |
|  | Male | 48.5 | 22.7 [21.1-24.3] | 0.778 |
|  | Female | 51.6 | 22.3 [20.8-23.9] |  |
|  | **Birth Order of Preceding Child**  First Birth | 53.1 | 21.9 [20.4-23.4] |  |
|  | Second Birth | 27.1 | 20.3 [18.3-22.4] | <0.001 |
|  | Third/Higher Birth | 19.9 | 26.9 [24.3-29.6] |  |
|  | **Survival of Preceding Child**  No | 6.3 | 56.9 [51.6-62.1] | <0.001 |
|  | Yes | 93.7 | 20.2 [19.1-21.3] |  |
|  | **Place of Residence**  Rural | 66.7 | 23.3 [21.9-24.6] | 0.057 |
|  | Urban | 33.3 | 21.0 [19.1-22.8] |  |
|  | **Migration Status**  No | 11.0 | 21.7 [18.4-25.1] | 0.639 |
|  | Yes | 89.0 | 22.6 [21.4-23.8] |  |
|  | **Region**  Eastern | 33.5 | 30.9 [28.7-33.0] |  |
|  | Central | 36.4 | 19.9 [18.1-21.6] | <0.001 |
|  | Western | 30.0 | 16.3 [14.5-18.1] |  |
|  | **Total** | **n = 5,441** | **22.5 [21.4-23.6]** |  |
| 244 |  |  |  |  |

245 The key independent variable in this study is women’s 3E. From Table 3, it is observed
246 that more than one-third of the respondents (39.1%) have coverage of below 50% in achieving
247 women’s 3E. However, 30.9% and 23.9% of women are found to have coverage 50%-75% and
248 75%-100%, respectively. It has been also found that only 6.0% of women have full coverage in
249 this regard. Table 3 also reveals that more than half (56.5%) of the women have illiterate
250 husbands. Moreover, more than two-thirds (72.9%) of the respondents got married off	at or
251 before the age of 18 years, while only 27.1% of them were over 18 years at the time of their
252 marriage. It has been also found that 20.8% of the women had experienced terminated pregnancy
253 in their lifetime, whereas, majority of them had not confronted such experiences. Furthermore,
254 40.2% of mothers are not exposed to mass media like radio, television, or newspaper, whereas
255 59.8% have access to them. Most of the respondents (92.6%) in the sample belong to the Muslim
256 religion, while a few of them (7.4%) are non-Muslims. Results from Table 3 also show that
257 19.2% of the women have a positive attitude towards intimate partner violence, whereas 80.8%
258 of them have not. Moreover, it is also found that 45.2% of women are employed, while 54.8%
259 are not. However, the proportions of male and female index children are almost equal in the
260 sample. For the case of birth order number of preceding child, it can be observed that most of
261 the children (53.1%) in the sample are the first births of their mothers, whereas, 27.1% and 19.9%
262 of them have birth order number two, and greater than two, respectively. It has also been
263 specified that 6.3% of children who are older between two consecutive births, have experienced
264 death, while 93.7% are alive. Again, two-thirds of the respondents reside in rural areas, whereas,
265 the rest of them belong to urban areas. However, the sample includes 89.0% of the women who
266 are migrants, while 11.0% are not. In addition, 33.5%, 36.4%, and 30.0% of the respondents are
267 from the eastern, central, and western regions of Bangladesh, respectively.
268 Table 3 reveals that 22.5% of birth intervals are in the short interval. The prevalence of
269 short birth interval is highest (27.1%) among the women who possess less than 50% coverage of
270 women’s 3E. An expected direction between short birth interval and coverage levels of women’s
271 3E has been observed that women with a higher coverage level of 3E tend to have a lower chance
272 of having short birth interval. Results show that the percentage of short birth interval for the
273 women with coverage of 50%-75%, 75%-100%, and 100% are 21.4, 18.3, and 14.9, respectively.
274 However, the p-value<0.001 presented in Table 3 provides enough evidence to justify women’s
275 3E to be a powerful tool that plays a significant role to control the risk of having a short birth
276 interval.
277 Table 3 also discloses the percentages of short birth intervals along with their 95%
278 confidence intervals for the categories of other selected variables. Results from p-values confirm
279 that the variables husband's education, maternal age at marriage, pregnancy termination, media
280 exposure, employment status, birth-order of preceding child, survival of preceding child, place
281 of residence, and region have a significant unadjusted association with short birth interval.

# 283 Sequential logistic regression models

284 The study analyzes the data using the sequential approach of the logistic regression model in
285 order to find out the association of women’s 3E with short birth interval in Bangladesh. To serve
286 this purpose, four separate models have been entailed where the first model (Model I) involves
287 only women’s 3Es as covariate. The second model (Model II) considers some women related
288 variables along with women’s 3E to examine how these women related variables change the
289 magnitude of association between women’s 3E and short birth interval. Similarly, the third
290 (Model	III)	model	extends Model	II by incorporating	some	variables	related	to child
291 characteristics. Lastly, the final model (Model IV) encompasses all covariates from Model III
292 with the addition of some household related covariates. Table 4 presents the estimates of the
293 parameters, odds ratios along with confidence intervals and p-values for all the four models.

# 296 Table 4. Odds ratio (OR) with 95% confidence intervals (CI) obtained from sequential binary logistic regression models

**OR with 95% CI**

| **Characteristics** | **Model I** | **Model II** | **Model III** | **Model IV** |
| --- | --- | --- | --- | --- |
| **Women’s 3E** |  |  |  |  |
| Coverage of < 50% | 1.00 | 1.00 | 1.00 | 1.00 |
| 50% - 75% | 0.74*** [0.63 - 0.86] | 0.74*** [0.63 - 0.87] | 0.76** [0.65 - 0.90] | 0.77** [0.65 - 0.91] |
| 75% - 100% | 0.61*** [0.51 - 0.72] | 0.55*** [0.45 - 0.67] | 0.56*** [0.46 - 0.69] | 0.59*** [0.48 - 0.73] |
| Full Coverage (100%) | 0.47*** [0.34 - 0.65] | 0.55** [0.39 - 0.77] | 0.56** [0.39 - 0.80] | 0.58** [0.41 - 0.83] |
| **Maternal Age at Marriage** (in years) |  |  |  |  |
| 10 - 15 |  | 1.00 | 1.00 | 1.00 |
| 15 - 18 |  | 1.31** [1.12 - 1.54] | 1.38*** [1.17 - 1.63] | 1.25** [1.05 - 1.48] |
| 18+ |  | 1.73*** [1.45 - 2.08] | 1.84*** [1.52 - 2.21] | 1.54*** [1.27 - 1.86] |
| **Husband’s Education** |  |  |  |  |
| Uneducated |  | 1.00 | 1.00 | 1.00 |
| Educated |  | 0.90 [0.78 - 1.05] | 0.93 [0.79 - 1.08] | 0.93 [0.80 - 1.08] |
| **Pregnancy Termination** |  |  |  |  |
| No  Yes |  | 1.00  0.86^+^ [0.74 – 1.02] | 1.00  0.84* [0.71 - 0.99] | 1.00  0.85^+^ [0.72 – 1.01] |
| **Media Exposure** |  |  |  |  |
| No |  | 1.00 | 1.00 | 1.00 |
| Yes |  | 0.93 [0.80 - 1.07] | 0.93 [0.81 - 1.08] | 0.98 [0.85 - 1.14] |
| **Religion** |  |  |  |  |
| Muslim |  | 1.31* [1.01 - 1.70] | 1.35* [1.03 - 1.77] | 1.40* [1.06 - 1.83] |
| Non-Muslim |  | 1.00 | 1.00 | 1.00 |
| **Employment Status** |  |  |  |  |
| No |  | 1.00 | 1.00 | 1.00 |
| Yes |  | 0.72*** [0.63 - 0.83] | 0.71*** [0.61 - 0.81] | 0.80** [0.69 - 0.92] |
| **Birth Order of Preceding Child** |  |  |  |  |

| First Birth | 1.00 | 1.00 |
| --- | --- | --- |
| Second Birth | 0.89 [0.76 - 1.05] | 0.85 [0.72 – 0.99] |
| Third/Higher Birth | 1.16* [0.97 - 1.38] | 1.03 [0.86 - 1.23] |
| **Survival of Preceding Child** |  |  |
| No | 1.00 | 1.00 |
| Yes | 0.19*** [0.15 - 0.24] | 0.18*** [0.15 - 0.23] |
| **Place of Residence** |  |  |
| Rural |  | 1.00 |
| Urban |  | 0.96 [0.83 - 1.12] |
| **Region**  Eastern |  | 1.69*** [1.44 - 1.98] |
| Central |  | 1.00 |
| Western |  | 0.86 [0.73 - 1.04] |

297	*Note*: ^+^p < 0.10; *p < 0.05; **p < 0.01; ***p < 0.001.

298 Results from Table 4 for Model I confirm a significant unadjusted association of
299 women’s 3E with short birth spacing. It has been observed that the odds of having a short birth
300 interval decreases by 26%, and 39% for women who have 50% -75%, and 75%- 100% coverage
301 of women’s 3E, respectively compared to those with coverage level less than 50%. Again,
302 women with full coverage of 3E have 53% lower odds of experiencing a short birth interval
303 compared to women having <50% coverage of 3E.
304 Results obtained from Model II show significant negative relationship between women’s
305 3E and short birth interval. It has been found that mothers who assure 50% -75% , 75%- 100%,
306 and 100% coverage of women’s 3E respectively, are 26%, 45%, and 45% less likely to
307 experience a short birth interval compared to those with 50% or lower coverage of 3E. Result
308 also confirms maternal age at marriage, pregnancy termination, religion, and employment status
309 to have a significant association with short birth interval. It has been found that mothers whose
310 age at marriage is 15-18, and 18+ have respectively, 31% and 73% higher odds of having a short
311 birth interval compared to the women aged between 10 to 15 years at the time of their marriage.
312 Again, mothers who experienced termination of pregnancy have 14% lower odds of having a
313 short birth spacing compared to those who did not. Moreover, Muslim women are 31% more
314 likely to deal with short birth spacing compared to non-Muslim women. Furthermore, employed
315 mothers have 28% lower odds of giving birth with a short interval compared to unemployed
316 mothers. However, Model II also exhibits that the husband’s education level and media exposure
317 have no significant association with birth interval.
318 The association between women’s 3E and length of birth interval has been analyzed in
319 Model III after adjusting for some women, and child related variables. Likewise, the previous
320 two models, Model III also provides enough evidence to justify a significantly strong association
321 of women’s 3E and short birth interval. It has been observed that the odds of experiencing a
322 short birth interval reduces by 24%, 44%, and 44%, when mothers possess 50% -75%, 75%-
323 100%, and 100% coverage of women’s 3E, respectively. According to Model III, there are 38%,
324 and 84% higher odds of having a short birth interval for women who got married at the age of
325 15-18, and 18+ years compared to their counterpart with age at marriage of 10-15 years.
326 Moreover, the odds of short birth interval for mothers who experienced terminated pregnancies
327 reduces by 16% compared to mothers who had no such experiences. Again, Muslim women have
328 35% higher odds of short birth spacing compared to non-Muslim women. Furthermore,
329 employed mothers are found to have 29% lower odds of a short birth spacing compared to
330 unemployed mothers. In addition, children with birth order number four or more are likely to
331 have 16% higher odds to be born with a short birth interval compared to the subsequent baby of
332 the first child. However, for a mother having a previous child alive, the odds of short birth
333 interval is 81% lower compared to a mother having lost the previous child. Likewise, Model II,
334 Model III does not show any significant association of husband’s education and media exposure
335 with short birth interval.
336 Model IV synthesizes all the covariates considered in model III with an addition of
337 women’s household characteristics. After controlling those important women, child and
338 household related variables, women’s 3E remains consistent to retain a significant association
339 with short birth interval. Results reveal that the odds of short birth interval decreases by 23%,
340 41%, and 42% with women’s 50%-75%, 75%-100% and 100% coverage level of 3E,
341 respectively compared to women with less than 50% coverage. That is women 3E plays a vital
342 role to minimize the risk of short birth interval. Moreover, women who got married at the age of
343 15-18, and 18+ years have respectively 25%, and 54% higher odds of short birth interval than
344 those aged 10-15 years. Moreover, mothers who experienced a terminated pregnancy in their
345 lifetime have 15% lower odds of short birth interval than mothers who did not. Furthermore,
346 women who belong to Muslim families are supposed to have 40% higher odds of occurring short
347 birth interval than non-Muslim women. Again, employed mothers are 20% less likely to
348 experience a short birth interval compared to unemployed mothers. Moreover, mothers having
349 the previous child alive have 82% lower odds of short birth interval compared to those with a
350 dead child. In addition, women who reside in eastern, and western regional areas of Bangladesh
351 have respectively, 69% higher, and 14% lower odds of short birth interval compared to mothers
352 who originated from the central areas of Bangladesh. However, husband’s education, media
353 exposure, birth order number of preceding child, and place of residence have no significant
354 association with birth spacing.

# 356 Discussion

357 Over the last two decades, researchers have paid much attention to study women’s status
358 focusing extra care towards women’s empowerment, educational level, and economic status
359 because of their significant contribution to different aspects of development including
360 reproductive health, worldwide. However, the study aimed to probe into the influence of the
361 power of these three socio-economic factors together, presented in this paper as women’s 3E
362 index, on the current scenario of short birth interval in Bangladesh. To serve this purpose, a
363 secondary data, extracted from BDHS, 2017-18 data, have been analyzed where the births of
364 reproductive women preceding five years of the survey are considered excluding those births of
365 non-pregnant mothers who have one child. This study showed that the overall prevalence of
366 short birth interval is 22.5% in recent years. That is, more than one in every 5 children is born
367 with a short birth interval in Bangladesh. This finding is consistent with the findings from
368 Rwanda (20%), Cameroon (21.3%) and Uganda (25.3%) [69]. Compared to Bangladesh, the
369 prevalence of short birth interval is higher in Iran (28.5%) and Ethiopia (45.8%) [59, 65].
370 Previous study of Bangladesh using data from BDHS, 2014 found the prevalence of 24.6%,
371 which suggests a poor declining trend in short birth spacing compared to the current study [20].
372 However, this is a matter to be concerned about because those children born with a short birth
373 interval are likely to experience early neonatal death [70]. Moreover, mothers who gave births
374 before the recommended optimal birth interval carry an increased risk of developing pregnancy
375 and health complications [70]. Although the proportion of short birth interval has decreased since
376 2014, our desired TFR is yet to be achieved.
377 In the bivariate analysis, the unadjusted associations of women’s 3E have been analyzed
378 and the result signifies a strong association between short birth interval and 3E. It has been
379 observed that the proportion of having short birth interval is higher for women who have less
380 coverage in achieving 3E. In regression analysis, sequential approach of logistic regression
381 model has been utilized to find out the adjusted association of women’s 3E index with short birth
382 spacing and to observe how these relationships behave while other important covariates related
383 to women, child, and household characteristics acted sequentially to the model. Findings show
384 that at each step, women’s 3E remains a significant factor with similar direction and magnitude
385 and thereby, contributes significantly to control over the choice of birth interval.
386 Analyzing the four models, it has been concluded that women’s 3E index has a strong
387 impact on the practice of short birth interval in Bangladesh. Hence, women empowerment,
388 education, and wealth index can be jointly considered as a potential force to influence the
389 likelihood of having a short birth interval. However, women empowerment has the highest
390 contribution to ensure the 3E of women, as reported in this study. Moreover, female education,
391 and economic status have almost the same contribution to women’s 3E index. Therefore, these
392 three indicators can be considered as influential determinants of short birth interval. Results
393 reveal a negative association between women’s 3E and short birth interval. That is, a woman is
394 less likely to experience a short birth interval when she is exposed to the higher coverage of
395 women’s 3E. Hence, women who are empowered, educated, and have higher economic status
396 are less likely to experience a short birth interval. To be specific, women empowerment has a
397 negative association with short birth interval. This finding is in tune with some previous works
398 conducted in other countries [48, 71]. Therefore, this is the high time to ensure women to be
399 more empowered because according to the study, empowered women have access to various
400 household-related decisions and thereby, can involve themselves in decisions regarding birth
401 interval behaviors to some great extent. Some studies proposed that in less developed countries,
402 lower fertility rates can be achieved by enhancing gender equity [72, 73]. This is because the
403 magnitude to which women start practicing equal rights with men, can contribute their opinion
404 with freedom regarding opting different family planning programs which in return helps in
405 deciding desirable family size.
406 Educating women is another pivotal approach to alleviate the risk of short birth interval
407 by means of attaining the power of women’s 3E, as demonstrated in this study. Some other
408 studies also figured out the importance of educating women to minimize the length of birth
409 interval [45-47, 74]. This could be because of the well-established fact that educated women are
410 more likely to have the knowledge regarding adverse health outcomes of short birth spacing and
411 therefore they are conscious enough on the use of modern contraception to lengthen their birth
412 intervals than uneducated women [75]. At the same time, the higher education level of women
413 is associated with getting better job opportunities which leads women to work outside their home
414 [76]. As a result, it may let women choose a limited number of children which consequently
415 lengthens spacing between births.
416 According to the study, women with higher economic status act positively to attain
417 greater coverage by assuring 3E of women. Therefore, it can be concluded that the likelihood of
418 having a short birth interval is lower for women who are economically rich which is consistent
419 to the findings of some previous studies [65, 74, 77]. A recent study conducted under some rural
420 areas in Bangladesh also showed that socio-economically disadvantageous women are more
421 likely to have a short birth interval [20].The reason behind this finding could probably be the
422 fact that the access to health knowledge, family planning, and maternal health care services for
423 women is getting enhanced with the increase in their economic status. Besides it was
424 demonstrated by a recent study that the higher socioeconomic status is associated with the
425 increased use of modern contraceptives which consequently reduces the chance of having the
426 next child and thereby lengthens the spacing between two successive births [78]. In addition, the
427 study has brought some secondary findings. The maternal age at marriage, experience of
428 terminated pregnancy, religion, employment status, survival status of previous child, and region
429 are found to have a significant adjusted association with birth interval.

# 430 Limitations

431 This study utilized the cross-sectional data extracted from BDHS, 2017-18 data which restricts
432 the scope to analyze the causal relationship between women’s 3E and short birth interval.
433 Moreover, the study did not take into account some other important control variables due to
434 absence in data. In addition, recent births born in the five years prior to BDHS, 2017-18 has been
435 considered that may create a recall bias in the study.

# 436 Conclusion

437 Women empowerment, education, and economic status can be jointly considered as a power of
438 women’s 3E, which is a vigorous element to play a highly significant role to control over the
439 choice of birth interval, as evident from the study. Therefore, increasing the status of women by
440 elevating educational attainment, economic conditions, and allowing them to actively participate
441 in household decision-making would help lessen the fertility rate in Bangladesh. To sum up, in
442 order to address the desired TFR and prevent intricacies of health outcomes for both mother and
443 child, an urgent need arises to initiate necessary strategies in this regard. These strategies should
444 include programs for the expansion of women’s educational opportunities and promotion	of
445 frequent family planning counseling to those who are economically less stable, and also to
446 encourage women to feel free to discuss any fertility related issues, especially with their spouses.
447 Although timing and spacing of pregnancy is a matter of choice for couples based on their
448 personal preferences and situation, it is a responsibility for those involved in health care to reach
449 the message of the optimal birth interval to general people to prevent adverse health and survival
450 outcomes of the mother and newborn.

# 451 Acknowledgments

452 We would like to thank the DHS Program and NIPORT, Bangladesh for allowing us to use the
453 BDHS, 2017-18 data for the current study.

# 454 References

455 1.	World Health Organization. Report of a WHO technical consultation on birth spacing: Geneva,
456 Switzerland 13-15 June 2005. World Health Organization, 2007.
457 2.	Tesema GA, Worku MG, Teshale AB. Duration of birth interval and its predictors among
458 reproductive-age women in Ethiopia: Gompertz gamma shared frailty modeling. Plos one. 459	2021;16(2):e0247091.

460 3.	Rutstein SO. Effects of preceding birth intervals on neonatal, infant and under‐five years mortality
461 and nutritional status in developing countries: evidence from the demographic and health
462 surveys. International Journal of Gynecology & Obstetrics. 2005;89:S7-S24.
463 4.	Fotso JC, Cleland J, Mberu B, Mutua M, Elungata P. Birth spacing and child mortality: an analysis
464 of prospective data from the Nairobi urban health and demographic surveillance system. Journal

465	of biosocial science. 2013;45(6):779-98.

466	5.	Conde-Agudelo A, Belizán JM. Maternal morbidity and mortality associated with interpregnancy

467	interval: cross sectional study. Bmj. 2000;321(7271):1255-9.

468	6.	De Jonge HC, Azad K, Seward N, Kuddus A, Shaha S, Beard J, et al. Determinants and consequences

469	of short birth interval in rural Bangladesh: a cross-sectional study. BMC pregnancy and childbirth. 470	2014;14(1):1-7.

471 7.	Kozuki N, Lee AC, Silveira MF, Victora CG, Adair L, Humphrey J, et al. The associations of birth
472 intervals with small-for-gestational-age, preterm, and neonatal and infant mortality: a meta-
473 analysis. BMC public health. 2013;13:1-9.
474 8.	Conde‐Agudelo A, Rosas‐Bermudez A, Castaño F, Norton MH. Effects of birth spacing on maternal,
475 perinatal, infant, and child health: a systematic review of causal mechanisms. Studies in family 476	planning. 2012;43(2):93-114.
477 9.	Erickson J, Bjerkedal T. Interval between pregnancies. The Lancet. 1979;313(8106):52.
478 10.	Klebanoff MA. The interval between pregnancies and the outcome of subsequent births. Mass
479 Medical Soc; 1999.
480 11.	Wendt A, Gibbs CM, Peters S, Hogue CJ. Impact of increasing inter‐pregnancy interval on maternal
481 and infant health. Paediatric and perinatal epidemiology. 2012;26:239-58.
482 12.	Li D-K, Wi S. Changing paternity and the risk of preeclampsia/eclampsia in the subsequent
483 pregnancy. American journal of epidemiology. 2000;151(1):57-62.
484 13.	Conde-Agudelo A, Belizán JM, Norton MH, Rosas-Bermúdez A. Effect of the interpregnancy
485 interval on perinatal outcomes in Latin America. Obstetrics & Gynecology. 2005;106(2):359-66.
486 14.	DeFranco EA, Seske LM, Greenberg JM, Muglia LJ. Influence of interpregnancy interval on
487 neonatal morbidity. American journal of obstetrics and gynecology. 2015;212(3):386. e1-. e9.
488 15.	Wijlaars L, Blackburn R, Gilbert R. Maternal vulnerability and birth interval in England: a
489 retrospective analysis of hospital episode statistics. International Journal of Population Data 490	Science. 2018;3(4).

491	16.	Barros FC, Bhutta ZA, Batra M, Hansen TN, Victora CG, Rubens CE. Global report on preterm birth

492	and stillbirth (3 of 7): evidence for effectiveness of interventions. BMC pregnancy and childbirth. 493	2010;10:1-36.

494	17.	King JC. The risk of maternal nutritional depletion and poor outcomes increases in early or closely

495	spaced pregnancies. The Journal of nutrition. 2003;133(5):1732S-6S.

496	18.	Razzaque A, Da Vanzo J, Rahman M, Gausia K, Hale L, Khan M, et al. Pregnancy spacing and

497	maternal morbidity in Matlab, Bangladesh. International Journal of Gynecology & Obstetrics. 498	2005;89:S41-S9.

499 19.	Rousso D, Panidis D, Gkoutzioulis F, Kourtis A, Mavromatidis G, Kalahanis I. Effect of the interval
500 between pregnancies on the health of mother and child. European Journal of Obstetrics and
501 Gynecology and Reproductive Biology. 2002;105(1):4-6.
502 20.	De Jonge HC, Azad K, Seward N, Kuddus A, Shaha S, Beard J, et al. Determinants and consequences
503 of short birth interval in rural Bangladesh: a cross-sectional study. BMC pregnancy and childbirth. 504	2014;14:1-7.

505	21.	Mayo Clinic Staff. Family planning: Get the facts about pregnancy spacing. Available at

506	https://[www.mayoclinic.org/healthy-lifestyle/getting-pregnant/in-depth/family-planning/art-](http://www.mayoclinic.org/healthy-lifestyle/getting-pregnant/in-depth/family-planning/art-20044072) 507	[20044072](http://www.mayoclinic.org/healthy-lifestyle/getting-pregnant/in-depth/family-planning/art-20044072). Accessed on May, 06/2021.

508 22.	Conde-Agudelo A, Rosas-Bermudez A, Norton MH. Birth spacing and risk of autism and other
509 neurodevelopmental disabilities: a systematic review. Pediatrics. 2016;137(5).
510 23.	Chen S-F, Shau W-Y, Hsieh C-C, Hsu J-J, Hung T-H. The impact of interpregnancy interval and
511 previous preterm birth on the subsequent risk of preterm birth. The Journal of the Society for
512 Gynecologic Investigation: JSGI. 2005;12(3):202-7.
513 24.	Cleland J, Bernstein S, Ezeh A, Faundes A, Glasier A, Innis J. Family planning: the unfinished 514	agenda. The Lancet. 2006;368(9549):1810-27.
515 25.	Lawn JE, Blencowe H, Pattinson R, Cousens S, Kumar R, Ibiebele I, et al. Stillbirths: Where? When?
516 Why? How to make the data count? The Lancet. 2011;377(9775):1448-63.
517 26.	Lawn JE, Blencowe H, Waiswa P, Amouzou A, Mathers C, Hogan D, et al. Stillbirths: rates, risk
518 factors, and acceleration towards 2030. The Lancet. 2016;387(10018):587-603.
519 27.	Alkema L, Chou D, Hogan D, Zhang S, Moller A-B, Gemmill A, et al. Global, regional, and national
520 levels and trends in maternal mortality between 1990 and 2015, with scenario-based projections
521 to 2030: a systematic analysis by the UN Maternal Mortality Estimation Inter-Agency Group. The 522	Lancet. 2016;387(10017):462-74.

523	28.	Ahmad I, Dewi YR. Birth Intervals and Infant Mortality in Indonesia. Population Review. 524	2020;59(1).

525 29.	Yohannes S, Wondafrash M, Abera M. Duration and Determinants of Birth Interval among Women
526 of Child Bearing Age in Lemo woreda, Hadiya Zone, SNNPR, Ethiopia. 2010.
527 30.	Rasheed P, Al Dabal B. Birth interval: perceptions and practices among urban-based Saudi Arabian
528 women. EMHJ-Eastern Mediterranean Health Journal, 13 (4), 881-892, 2007. 2007.
529 31.	Setty-Venugopal V, Upadhyay UD. Birth spacing: three to five saves lives. 2002.
530 32.	Exavery A, Mrema S, Shamte A, Bietsch K, Mosha D, Mbaruku G, et al. Levels and correlates of
531 non-adherence to WHO recommended inter-birth intervals in Rufiji, Tanzania. BMC pregnancy
532 and childbirth. 2012;12:1-8.
533 33.	Casterline JB, Odden C. Trends in inter-birth intervals in developing countries 1965-2014.
534 Population and Development Review. 2016:173-94.
535 34.	Greenspan A. Family planning's benefits include improved child health and nutrition: new data
536 from Bangladesh. 1993.
537 35.	Martin TC. Women's education and fertility: results from 26 Demographic and Health Surveys.
538 Studies in family planning. 1995:187-202.
539 36.	Ali FRM, Gurmu S. The impact of female education on fertility: A natural experiment from Egypt.
540 Review of Economics of the Household. 2018;16(3):681-712.
541 37.	Güneş PM. The impact of female education on fertility: Evidence from Turkey. 2013.
542 38.	Kanteh O, Palamuleni ME. Women status and fertility in The Gambia. Gender and Behaviour. 543	2019;17(3):13627-44.

544	39.	Atake E-H, Ali PG. Women’s empowerment and fertility preferences in high fertility countries in

545	Sub-Saharan Africa. BMC women's health. 2019;19:54.

546	40.	Samari G. First birth and the trajectory of women’s empowerment in Egypt. BMC pregnancy and 547	childbirth. 2017;17:1-13.

548	41.	Yadav AK, Sahni B, Jena PK. Education, employment, economic status and empowerment:

549	Implications for maternal health care services utilization in India. Journal of Public Affairs. 550	2020:e2259.

551	42.	Ahmed S, Creanga AA, Gillespie DG, Tsui AO. Economic status, education and empowerment:

552	implications for maternal health service utilization in developing countries. PloS one. 553	2010;5(6):e11190.

554	43.	Khan MT, Zaheer S, Shafique K. Maternal education, empowerment, economic status and child

555	polio vaccination uptake in Pakistan: a population based cross sectional study. BMJ open. 556	2017;7(3):e013853.

557	44.	Kabir MA, Khan M, Kabir M, Rahman M, Patwary M. Impact of woman's status on fertility and

558	contraceptive use in Bangladesh: Evidence from Bangladesh Demographic and Health Survey, 559	1999-2000. Journal of Family Welfare. 2005;51(1):1.

560	45.	Khan JR, Bari W, Latif AM. Trend of determinants of birth interval dynamics in Bangladesh. BMC 561	public health. 2016;16:1-11.

562	46.	Rabbi AMF, Karmaker SC, Mallick SA, Sharmin S. Determinants of birth spacing and effect of birth

563	spacing on fertility in Bangladesh. Dhaka University Journal of Science. 2013;61(1):105-10.

564	47.	Mahmood S, Zainab B. Analysis of birth interval in Bangladesh using product-limit estimate and

565	accelerated failure time regression model. Dhaka University Journal of Science. 2011;59(1):pp. 9- 566	15.

567 48.	Upadhyay UD, Hindin MJ. Do higher status and more autonomous women have longer birth
568 intervals?: Results from Cebu, Philippines. Social science & medicine. 2005;60(11):2641-55.
569 49.	UN	Inter-Agency	organizations.	Sustainable	Development	Goals.	Available	at
570 https://sustainabledevelopment.un.org/content/documents/11803Official-List-of-Proposed-
571 SDG-Indicators.pdf. Accessed on May, 06/2021. .
572 50.	World Bank. World Development Report 2012: Gender Equality and Development. World Bank;
573 Washington, DC: 2012. Available at https://openknowledge.worldbank.org/handle/10986/4391.
574 Accessed on May, 06/2021.
575 51.	National Institute of Population Research and Training (NIPORT), and ICF. 2020. Bangladesh
576 Demographic and Health Survey 2017-18. Dhaka, Bangladesh, and Rockville, Maryland, USA:
577 NIPORT and ICF.
578 52.	National Institute of Population Research and Training (NIPORT), Mitra and Associates, and ICF
579 International. 2013. Bangladesh Demographic and Health Survey 2011. Dhaka, Bangladesh and
580 Calverton, Maryland, USA: NIPORT, Mitra and Associates, and ICF International.
581 53.	National Institute of Population Research and Training (NIPORT), Mitra and Associates, and ICF
582 International. 2016. Bangladesh Demographic and Health Survey 2014. Dhaka, Bangladesh, and
583 Rockville, Maryland, USA: NIPORT, Mitra and Associates, and ICF International.
584 54.	Upadhyay UD, Gipson JD, Withers M, Lewis S, Ciaraldi EJ, Fraser A, et al. Women's empowerment
585 and fertility: a review of the literature. Social science & medicine. 2014;115:111-20.
586 55.	World Health Organization. Service availability and readiness assessment (SARA): an annual
587 monitoring	system	for	service	delivery:	reference	manual.	2013.
588 https://[www.who.int/healthinfo/systems/SARA_Reference_Manual_Full.pdf](http://www.who.int/healthinfo/systems/SARA_Reference_Manual_Full.pdf).
589 56.	Sen KK, Nilima S, Khokan MR. Readiness of Health Facilities to Provide Child Curative Care Services:
590 Evidence Based on Bangladesh Health Facility Survey, 2014. The Dhaka University Journal of 591	Science. 2020;68(1):29-35.
592 57. Cronbach’s Alpha: Simple Definition, Use and Interpretation. Available at
593 https://[www.statisticshowto.com/probability-and-statistics/statistics-definitions/cronbachs-](http://www.statisticshowto.com/probability-and-statistics/statistics-definitions/cronbachs-alpha-spss/)
594 [alpha-spss/.](http://www.statisticshowto.com/probability-and-statistics/statistics-definitions/cronbachs-alpha-spss/) Accessed on May, 06/2021.
595 58. Yohannes S, Wondafrash M, Abera M, Girma E. Duration and determinants of birth interval among
596 women of child bearing age in Southern Ethiopia. BMC pregnancy and childbirth. 2011;11:1-6.
597 59. Fallahzadeh H, Farajpour Z, Emam Z. Duration and determinants of birth interval in Yazd, Iran: a
598 population study. Iranian journal of reproductive medicine. 2013;11(5):379.
599 60. Hajian-Tilaki K, Asnafi N, Aliakbarnia-Omrani F. The patterns and determinants of birth interval in 600	multiparous women in Babol, northern Iran. Southeast Asian journal of tropical medicine and 601	public health. 2009;40(4):852.

602 61. Whitworth A, Stephenson R. Birth spacing, sibling rivalry and child mortality in India. Social science 603	& medicine. 2002;55(12):2107-19.

604 62. Aychiluhm SB, Tadesse AW, Mare KU, Abdu M, Ketema A. A multilevel analysis of short birth 605	interval and its determinants among reproductive age women in developing regions of Ethiopia. 606	PloS one. 2020;15(8):e0237602.

607 63. Brown W, Ahmed S, Roche N, Sonneveldt E, Darmstadt GL, editors. Impact of family planning 608	programs in reducing high-risk births due to younger and older maternal age, short birth intervals, 609	and high parity. Seminars in perinatology; 2015: Elsevier.

610 64. Pimentel J, Ansari U, Omer K, Gidado Y, Baba MC, Andersson N, et al. Factors associated with short 611	birth interval in low-and middle-income countries: a systematic review. BMC pregnancy and 612	childbirth. 2020;20:1-17.

613 65. Shifti DM, Chojenta C, Holliday EG, Loxton D. Socioeconomic inequality in short birth interval in 614	Ethiopia: a decomposition analysis. BMC public health. 2020;20:1-13.

615 66. Aleni M, Mbalinda S, Muhindo R. Birth Intervals and Associated Factors among Women Attending 616	Young Child Clinic in Yumbe Hospital, Uganda. International journal of reproductive medicine. 617	2020;2020.

618	67.	Suwal JV. Socio-cultural dynamics of birth intervals in Nepal. Contrib Nepal Stud. 2001;1:11-33. 619	68.	Mahmood S, Zainab B, Latif AM. Frailty modeling for clustered survival data: an application to 620	birth interval in Bangladesh. Journal of Applied Statistics. 2013;40(12):2670-80.

621 69. Chirwa TF, Mantempa JN, Kinziunga FL, Kandala JD, Kandala N-B. An exploratory spatial analysis 622	of geographical inequalities of birth intervals among young women in the Democratic Republic of 623	Congo (DRC): a cross-sectional study. BMC pregnancy and childbirth. 2014;14:1-10.

624 70. Nisha MK, Alam A, Islam MT, Huda T, Raynes-Greenow C. Risk of adverse pregnancy outcomes 625	associated with short and long birth intervals in Bangladesh: evidence from six Bangladesh 626	Demographic and Health Surveys, 1996–2014. BMJ open. 2019;9(2):e024392.

627 71. Nath DC, Land KC, Goswami G. Effects of the status of women on the first-birth interval in Indian 628	urban society. Journal of Biosocial Science. 1999;31(1):55-69.

629 72. Torr BM, Short SE. Second births and the second shift: A research note on gender equity and 630	fertility. Population and Development Review. 2004;30(1):109-30.

631 73. McDonald P. Societal foundations for explaining low fertility: Gender equity. Demographic 632	research. 2013;28:981-94.

633 74. Ajayi AI, Somefun OD. Patterns and determinants of short and long birth intervals among women 634	in selected sub-Saharan African countries. Medicine. 2020;99(19).

635 75. Shapiro D, Tambashe BO. Education, employment, and fertility in Kinshasa and prospects for 636	changes in reproductive behavior. Population Research and Policy Review. 1997;16(3):259-87.

637 76. Yousefy A, Baratali M. Women, employment and higher education schoolings. Procedia-Social and 638	Behavioral Sciences. 2011;15:3861-9.

639 77. Regasa Z, chakilu Tilahun B, Mesfin A. Spatial Distribution And Determinant Factors Of Birth 640	Interval Among Reproductive Age Group Women, Based On Edhs 2016, Ethiopia, 2019. 2020.

641 78. Nasrullah M, Zakar R, Krämer A. Effect of child marriage on use of maternal health care services 642	in Pakistan. Obstetrics & Gynecology. 2013;122(3):517-24.

643

644

Figure	[Click here to access/download;Figure;Figure 1.docx](https://www.editorialmanager.com/pone/download.aspx?id=28988522&guid=58750b13-7c2a-4932-bb31-3a5749b5b246&scheme=1)

Households (20,160)

Reproductive women (20,127)

Non-pregnant women having at least 2 or pregnant women having at least 1 live births born in preceding 5 years of survey

(4,866)

Pregnant women having at least 1 birth (346)

Non-pregnant women having at least 2 births

(4,520)

Pregnancies + Previous Births (514)

Last births (4,471)

Second last births (437)

Third last births

(19)

Total selected births in the study (5,441)

Births (4,927)

# Figure 1: Flow chart to select live births in computing birth intervals
