# Supplementary material for: Can women’s 3E index impede short birth interval? evidence from Bangladesh Demographic and Health Survey, 2017–18
Source: PLoS One. 2022 Jan 26;17(1):e0263003. doi: 10.1371/journal.pone.0263003 (PMC8791508; doi:10.1371/journal.pone.0263003)
Supplement: Attachment — Submitted filename: Response_Rev2.docx [file pone.0263003.s003.docx]

**Response to reviewer 2**

Comment 1: “Abstract: Background: in the Abstract part well written and structured but in your paper abstract background part express a number of studies found the significant association of women’s 3E in maternal health outcomes, but no studies, to the best of knowledge, have been found to justify the joint influence of women’s 3E on the birth interval. As several studies have revealed that the short birth interval increases the risk of adverse maternal, perinatal and infant outcomes and it is also responsible for increasing the country’s population size, more research is needed on the birth interval. The above two paragraph is controversial and in your manuscript page four starting from paragraph four and the first paragraph of page five talks is several study including Bangladesh justify the joint influence of women’s 3E on the birth interval so, why you say there is no studies, to the best of knowledge, have been found to justify the joint influence of women’s 3E on the birth interval?”

Response: Has been taken care of accordingly. Note that in literature, the variables women’s empowerment, education and economic status were used as covariates separately, whereas in this manuscript the joint effect of these three covariates was considered.

Comment 2: “Abstract: Conclusion: In Conclusion part you recommended Policy-making interventions are needed to raise awareness among uneducated, under- empowered and economically poor reproductive women through family planning and fertility control programs so that the country can achieve the desired fertility rate, so say Conclusion and recommendation.
”

Response: Has been adjusted accordingly.

Comment 2: “Method and Materials: Bangladesh Demographic and Health Survey (BDHS), 2017-18 data in Study design can you add reference of BDHS?”

Response: Reference was added.

Comment 3: “Method and Materials: table 1. Definition and measurements of the variables used in the study based on the 2017-18 BDHS: In the study, the education is converted into two categories: uneducated (no and primary), can we say primary education conclude as uneducated?

Response: Topics related to maternal and child health care is not included in the curriculum of primary level of education in Bangladesh. Therefore, we considered primary education level in the uneducated group.

Comment 4: “Method and Materials: what are your study participant’s inclusion and exclusion criteria?”

Response: Inclusion and exclusion criteria have been provided in Figure 1.

Comment 5: “Method and Materials: Statistical Analysis: what software uses to enter data and what software used to analysis data? Can you check goodness of fit tests? If you yes by what? You say Covariates having p-value less than 0.2 in the bivariate analysis were considered in the regression models. When you say statistically significant association independent and outcome variable? Please write detail in statistical analysis parts?”

Response: The name of the Software has been added on Page 13. The goodness of fit test has also been performed (provided on Page 19) and result is included in Table 4.

The reference has been included on Page 13 why p-value less than 0.2 was used. The p-value below 0.10 was used to define statistically significant association which was mentioned on Page 19.

Comment 6: “Method and Materials: Statistical Analysis: Explain how you went from OR to AOR, that is, what did you adjust for?”

Response: Note that in our manuscript, the term AOR was not used. The raised query in Comment 6 was addressed on Page 13 in Lines 223-224.

Comment 7: “The English must be improved. There are numerous errors. Some words start with a capital letter for no reason. Spelling errors can be corrected using Word spell check.”

Response: Has been taken care of. Microsoft word office 10 was used to prepare the draft.

Comment 9: “Update references with the most recent.”

Response: Has been updated.
