# Supplementary material for: Can women’s 3E index impede short birth interval? evidence from Bangladesh Demographic and Health Survey, 2017–18
Source: PLoS One. 2022 Jan 26;17(1):e0263003. doi: 10.1371/journal.pone.0263003 (PMC8791508; doi:10.1371/journal.pone.0263003)
Supplement: Attachment — Submitted filename: Reviewer comments PLOS ONE Final.docx [file pone.0263003.s001.docx]

Dear PLOS ONE editor

Thank you for giving me the opportunity to Review the manuscript titled

[**Can Women’s 3E Index Impede Short Birth Interval? Evidence from Bangladesh Demographic and Health Survey, 2017-18**].

After going through I see the manuscript detail, I forwarded my constructive comments which Authors missed to touch. Therefore, I am glad enough to express my sincerest thanks for giving me the opportunity to Review the manuscript. The Answers are marked in the yellow color. Thanks with regards.

1. Recommendation (Answer options):

Accept, Minor Revision, Major Revision, Reject)

2. Is the manuscript technically sound, and do the data support the conclusions? (Answer options):

(Yes, No, Partly)

3. Has the statistical analysis been performed appropriately and rigorously? (Answer options:

(Yes, No, I don’t know, N/A)

4. Does the manuscript adhere to the PLOS Data Policy? (Answer options :)

(Yes, No)

5. Is the manuscript presented in an intelligible fashion and written in Standard English? (Answer options: Yes, No)
6. Review Comments to the Author (minimum 100 characters)

**Comment #1: Abstract:** **Background:** in the Abstract part well written and structured but in your paper abstract background part express a number of studies found the significant association of women’s 3E in maternal health outcomes, but no studies, to the best of knowledge, have been found to justify the joint influence of women’s 3E on the birth interval. As several studies have revealed that the short birth interval increases the risk of adverse maternal, perinatal and infant outcomes and it is also responsible for increasing the country’s population size, more research is needed on the birth interval. The above two paragraph is controversial and in your manuscript page four starting from paragraph four and the first paragraph of page five talks is several study including Bangladesh justify the joint influence of women’s 3E on the birth interval so, why you say there is no studies, to the best of knowledge, have been found to justify the joint influence of women’s 3E on the birth interval?

**Comment #2: Abstract:** **Conclusion:** In Conclusion part you recommended Policy-making interventions are needed to raise awareness among uneducated, under- empowered and economically poor reproductive women through family planning and fertility control programs so that the country can achieve the desired fertility rate, so say Conclusion and recommendation.

**Comment #2:** **Method and Materials:** Bangladesh Demographic and Health Survey (BDHS), 2017-18 data in Study design can you add reference of BDHS?

# Comment #3: Method and Materials: table 1. Definition and measurements of the variables used in the study based on the 2017-18 BDHS: In the study, the education is converted into two categories: uneducated (no and primary), can we say primary education conclude as uneducated?

**Comment #4: Method and Materials:** what are your study participant’s inclusion and exclusion criteria?

**Comment #5: Method and Materials:** ***Statistical Analysis:*** *what* software uses to enter data and what software used to analysis data? Can you check goodness of fit tests? If you yes by what? You say Covariates having p-value less than 0.2 in the bivariate analysis were considered in the regression models. When you say statistically significant association independent and outcome variable? Please write detail in statistical analysis parts?

**Comment #6: Method and Materials:** ***Statistical Analysis:*** Explain how you went from OR to AOR, that is, what did you adjust for?

**Comment #7:** The English must be improved. There are numerous errors. Some words start with a capital letter for no reason. Spelling errors can be corrected using Word spell check.

**Comment #9:** Update references with the most recent

7. PLOS authors have the option to publish the peer review history of their article (<https://journals.plos.org/plosone/s/editorial-and-peer-review-process#loc-peer-review-history>). If published, this will include your full peer review and any attached files. Do you want your identity to be public for this peer review? (Answer options: Yes, No)
8. 7a. If you answered "Yes" above, that you would like your identity to be public, please provide your full name, as it should appear on the published peer review. Please do not sign the review on behalf of another person.

My Full Name:Dejen Getaneh Feleke

Confidential to Editor:

1. Do you have any potentially competing interests? If none, type "None." Our policy on competing interests can be found at <http://journals.plos.org/plosone/s/competing-interests>.

Answer: None

2. Did you receive any assistance in preparing this review (e.g. from a post-doc or graduate student)? If yes, please provide their name and title. If you have chosen to reveal your identity to the authors in question 7 above, please include the name of the person who assisted you in the response to question 7 as well. (optional)

Answer: No

***Yours sincerely,***

Dejen Getaneh Feleke

***Department of Pediatrics and Child Health Nursing, College of Health Sciences, Debre Tabor University, Debre Tabor, Ethiopia***
